# Barriers to prevention in oral health care for english NHS dental patients: a qualitative study of views from key stakeholders

**DOI:** 10.1186/s12903-023-03030-x

**Published:** 2023-05-27

**Authors:** Heather Leggett, Karen Vinall-Collier, Julia Csikar, Gail Veronica Ann Douglas

**Affiliations:** The School of Dentistry, The University of Leeds, Leeds, UK

**Keywords:** Prevention, Oral health, Qualitative, Behaviour change

## Abstract

**Background:**

Despite significant progress in the control of oral diseases since the discovery of fluoride in the 1940s, dental caries and periodontal diseases continue to affect a significant proportion of the population, particularly socially disadvantaged and lower socioeconomic groups. The National Health Service in England provides preventive advice and treatments as part of an oral health assessment, and evidence-based guidance recommends the use of fissure sealants and topical fluorides in addition to dietary and oral hygiene advice. Although oral health promotion and education have become expected parts of dental care, the need for restorative treatments remains relatively high. We aimed to explore how barriers to preventive advice and treatment for NHS patients may be hindering the provision of prevention in oral health to patients from the perspectives of multiple key stakeholders.

**Methods:**

Semi-structured interviews and focus groups were undertaken between March 2016-February 2017 with four groups of stakeholders: dentists, insurers, policy makers and patient participants. The interviews were analysed using deductive, reflexive thematic analysis.

**Results:**

Thirty-two stakeholders participated: 6 dentists, 5 insurers, 10 policy makers, and 11 patient participants. Four themes were developed: Perspectives on the clarity of oral health messaging and patient’s knowledge, The variability of prioritising prevention, Influences of the dentist-patient relationship on effective communication and Motivation to enact positive oral health behaviours.

**Conclusions:**

The findings from this research indicate that patients’ knowledge of and priority placed on prevention is variable. Participants believed that more targeted education could be valuable in enhancing these. A patient’s relationship with their dentist could also influence their level of knowledge through the information shared with them, their receptivity to the preventive messages and the value they place on it. However, even with knowledge, prioritising prevention and a good patient-dentist relationship, without motivation to engage in preventive behaviour the impact of these is reduced. Our findings are discussed in relation to the COM-B model of behaviour change.

**Supplementary Information:**

The online version contains supplementary material available at 10.1186/s12903-023-03030-x.

## Introduction

Public health systems and the oral health products industries have made important advances in the control of oral diseases since the discovery of fluoride in the 1940s [1]. Dramatic improvements in oral health have resulted in an increasingly dentate population, with many people retaining their teeth for life. Lifetime tooth retention is accompanied by a need to safeguard their health and prevent their deterioration with age. Dental caries and periodontal diseases are largely preventable through a combination of oral health self-care. This includes activities undertaken by the patient for example, twice daily toothbrushing with a fluoride toothpaste, and professional advice and interventions, for example instruction on how to maintain good oral health (healthy eating, toothbrushing, smoking cessation) and professional intervention such as application of fluoride varnish and prescribing high fluoride toothpaste [2]. Despite the impressive improvements in oral health, there is still much more to be done. In the case of dental caries, the most recent England-based survey of adult oral health indicated that almost one third of adults (27%) had untreated tooth decay [3], a similar picture to the 2009 decennial survey of adult oral health (n = 31%) [4]. For gum disease, 45% of adults had poor oral health with severe periodontal (gum) pocketing. It is important to note that as this survey is restricted to patients who attend dental practices, this sample of dental patients may well underestimate the clinical need in the true population. Furthermore, socially disadvantaged and lower socioeconomic groups are still more likely to experience a higher burden of oral disease (for example dental caries) [5, 6] and attend the dentist irregularly and symptomatically. This group were also under represented in the most recent England-based survey [3]. Within England, the National Health Service (NHS) provides for preventive advice and preventive treatments within a consultation as part of the patients’ oral health assessment [2, 7]. Evidence based guidance recommends effective preventive approaches including the use of fissure sealants and topical fluorides in addition to dietary and oral hygiene advice [2, 7]. Positively, results of the 2018 oral health survey of adults attending general dental practices suggests that preventive advice is provided by the dental team, with 89.5% of respondents reporting receiving some preventive advice [3]. Despite this, restorative treatment need has remained relatively high, with 90.2% of respondents having at least one filling.

Over the past three decades, oral health promotion and education have become part of the service expected by the General Dental Council to be delivered by the dental team [8]. Broadly, oral health prevention given by a dental professional can include applying fluoride varnish, placing fissure sealants, prescribing high–fluoride content toothpaste, providing oral hygiene advice and instruction, and providing dietary advice [2]. Oral health self-care undertaken by patients includes good oral hygiene practices such as regular toothbrushing, healthy diet/adjustment of lifestyle factors such as tobacco use and alcohol consumption, and regular dental attendance.

In order to improve the effectiveness of prevention for whole populations different approaches are needed that are based on sound evidence. Effective communication between the dentist and patient can be viewed as the cornerstone of a positive relationship [9]. Indeed, patient motivation and satisfaction are argued to be dependent on the dental team’s communication [10]. To aid effective communication, dental team members need to balance empathy and objectivity toward the patient with their treatment needs or care to promote oral health maintenance and improvement. Achieving this balance may be difficult as behaviour change conversations are challenging, especially when busy schedules and short consultation times [9] are factored in. Effective communication is hampered when discussions are not tailored to the patient’s needs, as information given can land badly and cause the patient to not feel valued, informed or listened to, this in turn is unlikely to motivate the patient to adopt advised behaviours [11, 12].

A critical appraisal of the literature in 2010 showed that [13] dentists felt that prevention was part of their professional responsibility, a source of job satisfaction, a marker of good quality care and as valuable to them as their practice image. In contrast, they felt that the dentist-patient relationship may be compromised when discussions with patients were of a sensitive nature (for example reducing alcohol intake) and questioned the relevance of dentistry to certain public health interventions such as smoking cessation [13].

There is a distinct lack of literature on dental patients’ opinions on prevention in oral health care, especially what ‘prevention’ means to them and what they perceive their role to be. Consequently, it is not clear to what extent dental patients accept preventive messages, how aware they are of what prevention is and its relevance to them and whether this leads to active behaviour change for them.

This qualitative study was one element of a larger EU funded, Horizon 2020 project – ADVOCATE [14]. Focus groups and interviews with key stakeholders were used to explore perception’s regarding the barriers and facilitators to prevention in general dental practice across 6 partner countries in the ADVOCATE project: England, Denmark, Netherlands, Hungary, Germany and Ireland. The key stakeholders interviewed were: dentists, insurers, policy makers and patients. Broad findings from this qualitative research have been previously published focusing on experiences from across all six countries [15]. Adopting a narrower focus, this article presents findings from the English data regarding barriers to preventive advice and treatment for patients. We aim to explore how these barriers may be hindering the provision of prevention in oral health to patients.

## Method

### Participants

Semi-structured interviews and focus groups were undertaken with dentists, policy makers, insurers and NHS patient participants. Patient participants were members of the general public who used NHS dental care and were not employed as one of the other stakeholders included in the study. Dental participants were dentists who were solely or partly employed as an NHS dentist. Insurers in this context were those involved in the commissioning of NHS primary care dental services. The policy makers were individuals who developed high quality advice for the general public and provided specialist advice to the government and health agencies. The majority worked within the civil service and were dentally trained before becoming consultants within dental public health. This advice and guidance forms policy and underpins service development and delivery. Although their role around policy is mainly advising rather than making, we have kept the terminology ‘policy makers’ to be in keeping with our previous published work on the wider EU dataset. As we were interested in prevention within NHS dentistry, we did not include any private dental insurers, private only dentists or private only dental patients.

The participants were mainly recruited using opportunity and purposeful sampling. The research team’s network was utilised to identify and contact dentists, policy makers and insurers; they were contacted individually to ask if they would like to participate. Due to the smaller pool of participants and their limited availability, only opportunity sampling was used to recruit insurers and policy makers. Patient and dentist participants were recruited through opportunity and purposeful sampling. The patient participants were identified through their involvement in a dental patient-public involvement (PPI) group at the University of Leeds and snowball sampling was used through this network to recruit other members of the general public. Efforts were made to ensure a varied age range, gender and socio-economic status within the patient group and a varied sample of NHS only and NHS and private dentists as well as equal representation of genders and time since qualification in the dentist group. In terms of geographical variation, the patient and dentist participants were from the Yorkshire region. The policy maker and insurer participants were from across England. Ethical approval was granted from the Dental Research Ethics Committee, University of Leeds (051115/HL/182).

### Materials

Topic guides were developed from a literature search on prevention in oral health care (Supplementary file 1) and refined using the research team’s knowledge and experience. The questions in each topic guide enabled an exploration of barriers and facilitators to prevention from each stakeholder’s unique perspective. For example, questions to the dental team asked about perceived barriers and facilitators to providing prevention to patients, whereas questions to the general public asked about whether they felt they received preventive advice and what aided and hindered this. Policy makers were asked what influenced policy, how they felt policy aided prevention in practice and how this could be improved. Insurers were asked how well they thought prevention was addressed within oral health care, what role they played within this, the barriers to providing more prevention and how it could be strengthened.

### Procedure

After initial contact had been made and the participant had agreed to participate, a time and date was agreed upon for the interview to take place. Before the interview or focus group began the participants were asked to re-read the information sheet and to read and sign the consent form. Participants were reminded that they could ask for the interview to be stopped at any time. Each interview or focus group lasted between 25 min and 1 h and were undertaken by an experienced qualitative researcher (HL) who was not previously known to the participants. The patient focus group took place at the University of Leeds. The policy makers, insurers, dentists, and one patient participant were interviewed either over the telephone or in person in their place of work.

### Analysis

The recorded interviews were transcribed verbatim. Any identifying information was removed from the transcripts and participants were given a pseudonym. The transcripts were imported into Nvivo to aid data management. Interviews were analysed using reflexive thematic analysis [16], which took a deductive approach since we had a pre-identified set of ideas and constructs we wanted to code around barriers and facilitators to prevention for patients. Consequently, the transcripts were read and coded by HL in relation to barriers and facilitators to giving and receiving prevention in oral health care. After initial coding an iterative process began of reviewing and revising the codes by HL and KVC. This led to the development and refinement of themes pertaining to barriers and facilitators to prevention in oral health care for patients. Regular research team meetings were held to discuss the codes and themes at key points during analysis. During analysis we found that patient participant’s views of themselves as patients were often contrasted with their perspectives of patients more broadly. As such, where we refer to the ‘patient participants’ in the results we are referring to the experiences of the patients we interviewed. Where we refer to ‘patients’ or the ‘general public’ we are referring to the opinions of the participants on patients in general. The following codes are used to represent each group: Dentists = DEN, Policy makers = PM, Insurers – INS and Patients = PAT.

## Results

Interviews took place between March 2016 and February 2017. There were thirty-two participants: 6 dentists, 5 insurers, 10 policy makers (4 in a focus group and 6 interviews), and 11 patient participants (10 in a focus group and 1 interview).

Four themes were developed: Perspectives on the clarity of oral health messaging and patient’s knowledge, The variability of prioritising prevention, Influences of the dentist-patient relationship on effective communication and Motivation to enact positive oral health behaviours. Due to the multifaceted nature of prevention and behaviour change, these themes are not mutually exclusive and are strongly interrelated with one another. Our analysis focused on barriers and facilitators to prevention, however in most instances a facilitator was the direct opposite of the barrier. We found that participants more naturally spoke about barriers and so our themes focus on and reflect key barriers to prevention for patients. Within each theme, the barriers to patients are described from the perspectives of all four groups of participants.

### Perspectives on the clarity of oral health messaging and patient’s knowledge

The patient participants we interviewed reported having a general awareness, knowledge and understanding of what prevention was and what it entailed *“I think personally I would say prevention is… just a number of things. Like your diet. And also em… ensuring that you are brushing your teeth twice a day.” [PAT10]*, as well as visiting the dentist regularly. However, thinking about the population more broadly, all participants believed that the general public in the wider population had little knowledge of how to look after their mouth and what constitutes a healthy oral hygiene regime.

*PM6: I remember when I was in practice, you know that there were patients who would brush their teeth once a week! And thought that that was kind of ok.*

It was agreed by participants from all stakeholder groups that there is a general lack of education and therefore knowledge for the public regarding prevention. It was felt that there should be more oral health campaigns such as healthy eating and that this would be a factor that could influence attitudes of the general public and enhance their knowledge of what good oral health care and prevention looked like. In addition, it was seen that patients were sometimes given mixed messages by dental teams, and they felt that there was a lack of clear take home messages for them. The patient participants often felt that there was a conflict between dentists’ recommendations and the messages given in advertising campaigns particularly around ‘healthy’ foods and drinks. One area of conflict was around the messaging for sugar. It was common knowledge that sugar caused tooth decay. However, the fact that this also extends to ‘healthy’ sugars such as fruit and fruit juices was often not conveyed to our patient participants or not understood by them.

*PAT10: I don’t know why my child’s got tooth decay!” And then you ask what do they eat? They, they eat sort of raisins and grapes and bananas and sweets! (Laughs) So maybe it’s, it’s more that … sweets are bad but they’re not the only sweet thing that’s causing… tooth decay.”*

*DEN5: But the message should be important. Very important thing and another document that will help you as well, the message coming out is always wrong. Because if you look at the tooth brushes tooth paste ads they will always have the tooth paste off the full ribbon…I’ve raised it so many times! And I’ve got it changed from a couple of places but not in the majority of places. So if you are having billboards on the buses that, I think they had it on the buses, it was a full ribbon again. So if you sit there, putting your heart out and telling them about pea size, they never know what pea size is because they always see this. Absolutely. So this is very important that mixed messages should not be there.”*

### The variability of prioritising prevention

The patient participants perceived prevention and oral hygiene messages as valuable and important. In addition, they wanted more preventive information and more information about how to look after their teeth. Patient participants saw toothbrushing as an important part of their daily routine. Despite its importance to them personally, some felt as though oral health care was given a low priority by the government; they believed that because dentistry is only partially funded by the NHS that this meant that it is not viewed as important as medical care is by the government.

*PAT.FG.P2: “Desert [island] discs…. I’ll take my toothbrush with me. That’s one I, I feel dirty if I don’t clean my teeth. So it’s something that I’ve always done and I think no matter what desperation, I would always, even if it was just salt! I’d want to rinse my mouth.”*

*PAT.FG.P3: “Cos the Government haven’t given it, it’s important. So if you’ve got a cough, get down to your GP!..... Got a bit of a sore tooth, well I can’t afford to do that this year, so I’ll, I’ll live with it!”*

From a dentist, insurer and policy maker perspective, patients were generally viewed as having a lack of awareness of what prevention was, not valuing it and not prioritising the actions that would help achieve it. The patient participants also discussed instances/experiences with friends or family that corroborated these views. Dentists witnessed a lack of value in prevention in patients who did not return for follow up appointments, or those who had not made any changes to their diet or oral hygiene since their last appointment. They believed that patients’ lack of knowledge combined with the low priority they placed on their oral health often meant that they didn’t recognise that there was a problem with their oral hygiene or lifestyle that needed addressing.

*DEN1: “You do think, but it just shows the, the general attitude of somebody out there who wasn’t, wasn’t really, you know, a person who was educationally subnormal. She knew but she just thought mango’s healthy. They also think Ribena’s healthy! Because, because of the fruit on the front!”*

*INS6: The barriers are peoples’ unawareness of simple things you can do to improve your oral health, their motivation to do that…”*

*PM3: “So you’ve got patients who, perhaps they believe their oral health is fine. They do, what they’re doing is quite sufficient so why would they change? Em… or it’s a very low priority in their lives. They’ve got lots of other things going on. And… em… no it’s not something they want to try to change. The ability to change at all.”*

Often, oral health was viewed not to be a priority for patients due to other important life issues being of greater importance to them; in terms of their hierarchy of needs, oral health care was seen to fall down the list. Those with chaotic lifestyles were viewed by all participants as being more likely to live in the moment, rather than looking ahead to the future and as such were less likely to think ahead with regards to their long-term oral health. Seeing a dentist was often seen as costly by patient participants; this was viewed to further negatively influence attitude toward prevention and oral health care, attendance at the dentist and their willingness to act upon any preventive advice given. Dentists and policy makers also believed that patients were unlikely to be willing to pay extra for preventive services because *“Patients don’t pay for advice! They generally pay for something active, something physical” [DEN1]*, and some of the patient participants agreed with this.

*PAT.FG.P6: Yes I mean if you’ve got no money it’s [dentistry] the last thing you want to spend your money on.”*

*DEN1: “I do find that… if we bring patients back, if we, if patients need 2 or 3 deep cleans they don’t feel they’re, they’re getting anything so they tend, a lot of them tend not to bother coming back because as I said, gum disease isn’t visible and it doesn’t hurt! You get bleeding gums but patients don’t care as long as it’s not bleeding when you’re talking! That’s it, they don’t … most of them don’t care cos they don’t recognise that there’s a problem!”*

*PM3: “Effectively pay to say… you know… I’m going to, I’m going to spend your time with you sitting there while I talk to you. And expect you to go away and do something. And at that point I’m going to charge you for the privilege.”*

Those with role models who do not prioritise their oral health or have good oral health regimes were also seen as being less likely to prioritise it and as more likely to just accept their oral health rather than try and change it, or even understand why having poor oral hygiene is a bad thing.

*PAT.FG.P6: “Cycles, you know cycles of poverty. Cycles of deprivation. Haven’t seen their parents doing it. Wasn’t important”*

*DEN3: “Definitely. And like I say without stereotyping (laughter) but you do tend to see it in certain pockets of society. It definitely, yes, cos the parents don’t come. They don’t know. They don’t know any better. It just gets… the children pick up the same thing. They don’t know any better.”*

As with knowledge, the dentist’s value of prevention is key to influencing whether they will provide preventive advice to patients. We found a pervasive attitude within dentistry that “*crowns, the bridges, it’s the restorative is dentistry” [DEN3].* It was common for dentists to believe that advising patients was not part of their job, it was not a priority to them and had less *“importance compared to paediatric, prosthodontics, orthodontics. I don’t think it gets the same credit. And I think that as students gets fed in quite a lot.*” [DEN3]. Such an attitude does not create or foster an environment whereby providing proper, effective preventive advice is a main concern of a dentist within a dental check-up.

### Influences of the dentist-patient relationship on effective communication

It was recognised by all participants that a poor relationship between a patient and their dentist could act as a barrier to them receiving preventive advice. It was important that the advice given and the preventive message shared was tailored to the individual as this made the patient feel more valued. It was recognised by policy makers and patient participants that the advice needed to be focused on something that was within the patient’s ability to change. Furthermore, providing the message in a positive way rather than making the patient feel as though they were being ‘told off’ was a facilitator to them being receptive to the preventive advice. The patient participants wanted to be included in all the knowledge and information surrounding their oral health and involved fully in the decision making process. Those who recalled feeling involved felt more empowered and thought they had a positive and happy relationship with their dentist. This also gave them the control to decide whether or not they were going to enact the advice.

*DEN2: It’s the relationship you build up with the patient often [that makes them receptive to prevention]… em… I mean if you’ve been seeing a patient for a while and they’ve got trust in you and the practice, they will start to believe what you’re saying. If you back it up with evidence and say that’s why we’re giving you this information as well, delivering oral health… It comes down to that. And I think with the sugar stuff it comes from celebrities, the power of celebrity endorsement as well saying reduce your sugar intake works*

*PAT.FG.P6: “Great acknowledgment of the fact you might be anxious. And sort of, you know and, and you, actually sitting with you before he does the treatment, to actually tell you what he’s going to do.”*

*PAT.FG.P2: I think sometimes they just have to be a bit more sort of… factual and not afraid to point out… You know the problems that eating too much sugar and what have you! I think sometimes dentists are a, feel a bit… fearful of actually lecturing too much!”*

The patient participants did voice some feelings of mistrust towards dentists; they felt as though the dentist was often more interested in making money than providing appropriate and comprehensive care. The patient participants saw dentists as not being interested in or prioritising prevention. Additionally, they believed that the dentist did not give them enough advice; they wanted full disclosure and more information from the dentist so that they could be fully involved in any decision making. Another barrier was the time constraints placed on their appointment; they often felt like the consultation was rushed and this made them feel less valued and thus negatively affected the dentist-patient relationship.

*PAT2: “No! I just think a dentist will rush a lot…..The orthodontist told me I needed to see the hygienist. Em I think there was some build up on my teeth. And so when I went for my check-up and I mentioned this to the dentist and now your teeth are fine. So then I actually don’t know are they? Or aren’t they? But then it was a rushed appointment and I feel the same thing for my children.”*

*PAT.FG.P7: “But now she just fills out this form like a robot every time I see her and just hands it to me! And there’s no discussion it’s just like it’s just a tick box exercise of I have to do this form! There you go, take that with you.”*

*PAT.FG.P6: “It’s to give information and be factual but not judgemental and, and dentists, generally the ones I’ve come across, don’t seem to be very good at that! Or they’re not practised at it.”*

Dentists’ voiced difficulties if they struggled to communicate with the patient due to cultural or language barriers. Dentists highlighted that this relationship was a reciprocal one, and that there was only so much they could do to engage a patient. It was agreed by all participants that regardless of how hard a dentist tried to build a relationship with the patient to enhance the messages being shared, if oral health prevention was not a priority for the patient, then they would not engage with any preventive advice offered.

### Motivation to enact positive oral health behaviours

For many, improvements in and maintenance of oral health requires behavioural regulation. Behavioural regulation will not occur or be sustained if the individual is not motivated to do so [2]. Consequently, a lack of motivation to enact or maintain a behaviour can be potential barriers to the provision of preventive care and patients acting on preventive advice given by the dental team [2, 17]. In order for patients to be motivated to engage in a sustained behaviour, they must view their oral health as a priority. As evidenced in the previous themes, prevention being a priority goes hand in hand with being knowledgeable about what prevention is and recognising its value. Without oral health knowledge or valuing this knowledge we found that patients were less likely to be motivated to enact positive oral health behaviours. Similarly, the experiences of our participants illustrated that the strength of the dentist-patient relationship can influence patients’ knowledge, value and ultimately their motivation.

Most of the patient participants perceived themselves to be receptive to preventive messages, valued the advice, wanted to be active in looking after their oral health and ultimately wanted to improve their oral health through sustained behaviour change. These individuals experienced positive reinforcement in seeing changes in their oral health both physically (being pain free) and cosmetically and felt as though this had helped them to form new habits.

*PAT.FG.P6: Yes. Yes absolutely. I’ve got better at my, how, cleaning my teeth you know? (after being shown by the dentist)”*

*PAT2: “Sometimes I feel as though I’m just too tired and then I don’t. And then when I start to see, like when I’m brushing my teeth and then I see some blood and think ok! I really need to make sure I brush my teeth.”*

*PAT.FG.P1: “Well I think it’s just a matter of learning your kids, to give them a toothbrush and some toothpaste and starting young.”*

However, it was recognised that not all patients are motivated to change their behaviour. Those lacking motivation were more likely to be those who do not take responsibility for their own oral health and don’t engage with preventive advice given.

*DEN1: “There are some patients who… em… aren’t that interested in the talking bit and they just want to get on and get out of there! Em but again I think that’s about abdication of responsibility for themselves so it’s about handing that responsibility back to people.”*

*DEN3: “…. Or their thing was every time I come to the dentist, it’s your job to clean my teeth! And they didn’t quite understand that it’s home care. You need to do it yourself! You need to get. And, and over there I mean they all got prescriptions of mouthwash and toothpaste. That was also one of the reasons they would come back cos they ran out at home. They don’t want to buy toothpaste for £2 or whatever!”*

*INS3: “So it’s not going to happen to me! When it does I think there’s also a denial and I think many parent initially feel guilty then it’s, well it’s normal isn’t it? Children, children get caries or you get holes in your teeth! So there’s a, we, there’s an acceptance, there’s an acceptance. So there’s that psycho-social barrier. How do we nudge society? And nobody wants to be told off. So we spent many years blaming patients. So I think we’ve got to move away from that sort of thing.”*

Participants discussed a number of barriers which were seen to negatively impact motivation to engage in sustained behaviour change. Commonly, patients may have a fear or phobia of attending the dentist. This might mean that they only attend when they are in pain, which makes delivering the preventive message to patients frequently more difficult. In addition, by attending only when they are in pain, patients are further fuelling their fear or phobia of the dentist as ‘invasive’ or ‘painful’ as it is more likely that treatments are required.

*PAT.FG.P4: “And I’ve got a 6 year old daughter. And I take her for em, every 6 months. Well every 3 months at the moment because she’s got bad anxiety with … dentists. So I’m taking her a little bit more and I feel quite bad because I should be leading an example you know? But it’s just because when I was younger I had braces. And they were like everything’s fine and touch wood I don’t have any fillings. I, I feel that if I go back now they might say you need fillings! I think that’s my fear.”*

*PAT.FG.P6: “I didn’t go to the dentist for 3 years because I just couldn’t face it. And then went to my husband’s private dentist and he has restorative dental treatment and so I went to use that! And yes it’s expensive!”*

*DEN4: “There’s something about a power shift as well. So… em… as a dentist with a drill in your hand, you’ve got your patient in the prone position with their mouth open very, very vulnerable and you’re having control over them. And that’s why a lot of patients don’t, that’s why there’s phobia thing about going to see a dentist cos you’re losing control.”*

In addition, some of the patient participants felt that they were not rewarded for their attempts to change their behaviour or that such infrequent contacts with the dental team meant that the preventive message was not reinforced enough to foster change. In terms of reinforcement, change was perceived to be easier if patients could see the consequences of their attempts. This was seen as a barrier by patients since, apart from less plaque and no bleeding they reported little feedback on whether they were doing a good job or not. Furthermore, change in itself was viewed to be difficult as it often required a long-term change in lifestyle (diet) not just more regular tooth brushing or flossing. A change in oral health related habits was seen as harder to achieve and maintain compared to other health issues where the benefits of the behaviour change could be evidenced more easily and quickly and thus provide ongoing positive reinforcement.

*PAT.FG.P2: “And I said well the point is we’re building this up now. She’s so scared she won’t even open her mouth! You know? Em and they said maybe next time when you open your mouth. So I thought… I’d have just you know.”*

*PAT.FG.P5: “I think there’s that initial guilt thing isn’t there? Where you, when they tell you that you’re doing something wrong and then you rush out and you buy the, the floss and you, you get the sticks and then maybe for the next 2 weeks or something you find that 5 minutes in your day to do your flossing regularly and then it sort of slowly edges off?”*

A further barrier was the view of visiting the dentist as a ‘transaction’; *you go there, you pay for something and you get something done to you*. [PM7]. This was seen to lessens patients’ motivations to enact take home ‘advice’ if they perceive that they are paying to receive a service from the dentist.

## Discussion

The findings from this research indicate that patients’ knowledge of and priority placed on prevention is variable and that participants believed that more targeted education was necessary. A patient’s relationship with their dentist could also influence their level of knowledge through the information shared with them, their receptivity to the preventive messages and the value they place on it. Finally, despite the importance of knowledge, prioritising and a good patient-dentist relationship, without motivation to engage in preventive behaviour the impact of these is reduced.

All stakeholders talked about and saw prevention as something relatively simple and easy to understand; however, the high rates of restorative treatments in England suggest that it still is not enacted by many patients[3]. And findings from a survey on key oral health messages suggest that dentists could be more informed than they are [18]. Our results suggest that the dentist-patient relationship and motivation to enact preventive behaviour could be the missing link between knowledge and value of prevention and preventive behaviours being enacted.

Knowledge is vital to patients changing their behaviour. The importance of knowledge has been highlighted by a number of oral health focused systematic reviews which show that knowledge can be improved through dental education given to patients including the provision of verbal oral health messages, or by the reading of written materials [10, 19, 20]. Knowledge is important because without it, a patient cannot begin to put in place necessary oral health changes. Educational interventions can promote changes in oral health behaviours such as: the use of dental services, oral hygiene and the reduction of sweet consumption [21]. There is less evidence for the improvement of periodontal outcomes such as plaque reduction and the incidence of new cases of caries [21]. Educational interventions appear to have short-term benefits, suggesting that the behaviours are not sustained over time [19, 20], supporting the claim that knowledge alone is unlikely to lead to behaviour change [10]. Knowledge can also be influenced by attitude; patients having a positive attitude toward prevention is related to an increased level of knowledge of periodontal health [22]. Across all our stakeholder groups, more education for patients was seen as important in order to increase their knowledge and prioritisation of prevention and to facilitate motivation to engage in preventive behaviours.

Previous research also supports the importance of the dentist-patient relationship, the vital role a dentist plays in supporting patients and the negative this can have on how patients feel [23–26]. Kay et al., (2016) [10] suggest that a patient’s acceptance of and subsequent action after receiving oral health promotion is dependent on the perceptions of the patient regarding their relationship to the message provider, and the provider’s understanding of the patient’s life and behaviour. Simply, if the patient cannot relate to the health professional, they are less likely to accept and act on the oral health advice they are given.

Oral health promotion which is provided based on behavioural and psychological models have been shown to be the most effective way to educate patients and encourage behaviour change [27]. Motivational interviewing in the clinical setting has been shown to have short-term positive impacts on patients’ health behaviours [28] with more recent studies showing improvements in oral health behaviours across a variety of patient groups (pregnant women, children, adolescents) [29–31]. However, traditional behaviour models such as the Theory of Planned behaviour and Stages of Change model have failed to bring about consistent oral health behaviour change in patients [17]. As such, the COM-B model and the associated behaviour change wheel of behaviour change techniques have gained traction with dental public health researchers as a means to understand and facilitate oral health behaviour change [32]. Newton and Asimakopoulu [32] outline three steps to delivering interventions aimed at improving oral health related behaviour based on the COM-B model [33] and the behaviour change wheel [34]: creating capability, enhancing motivation to change, and creating opportunities to enact the oral health behaviour and forming a habit. Behaviour change is seen to consist of these three components, and all are needed in order for sustained behaviour change to take place. These components link up with our findings to shed light on how barriers to prevention for patients could be addressed and overcome.

An individual’s capability to perform a behaviour can be enhanced by the provision of information and guidance about that behaviour. This links in with our findings around the importance of knowledge and value placed on preventive oral health behaviours as well as the role the dentist-patient relationship plays here. Newton and Asimakopoulu [32] suggest practical communication tips to support dental professionals delivering preventive oral health advice to patients including: ensuring the message is understandable from a lay perspective, emphasising and providing the most important information first, sending reminders and using specific statements. It is suggested that patient’s motivation can be enhanced by targeting cognitions likely to enhance that behaviour such as emphasising the benefits of behaviour change and providing information on the patient’s risk of oral disease [32]. These suggestions support our findings regarding the importance of the dentist-patient relationship in supporting the patient and the negative impact this can have when the relationship is poor. The patients we interviewed sometimes felt uninformed during their consultation or that the information given was not tailored to their needs. Adopting some of the communication and cognition strategies outlined above may help to facilitate the dentist-patient relationship and patient’s capability and opportunities to engage in behaviour change.

The next step outlined by Newton and Asimakopoulu surrounds creating opportunities for patients to put their motivation into action and addresses ways in which patients can be supported to transform their motivation into action. Strategies include setting goals with patients, setting plans for when, where and how a particular behaviour should occur and encouraging patient self-monitoring through paper or electronic diaries or record sheets. Finally, it is important that a habit is formed, otherwise long-term behaviour change will not occur and patients are less likely to see sustained positive oral health outcomes. Habit is aided by repetition of behaviour and patient self-monitoring and through this patients will begin to be cued to enact the oral health behaviour by their environment [32]. These suggested strategies could address some of the barriers participants discussed in the *Motivation to enact positive oral health behaviours* theme. Patient participants often reported not feeling supported enough by the dental team to change their behaviour due to the short duration and limited frequency of dental visits. Assisting patients in setting goals, planning and recording self-monitoring may help to overcome some of these barriers experienced by patients. This could be further reinforced through discussion with the dentist regarding the importance of regular dental attendance. Patients frequently found it difficult to see any changes as a result of their behaviours, this then negatively impacted their motivation to continue with the behaviour change. Self-monitoring and goal setting may help to motivate patients through prompting the behaviour, as a means to monitoring progress and to adjust their plans if necessary. Further supporting the role of self-monitoring, repetition and reinforcement in aiding habit forming, our patient participants who did see changes in their oral health as a result of their behaviour change experienced positive reinforcement which facilitated them in forming longer-term habits.

Whilst the strategies of creating capability, enhancing motivation to change and creating opportunities to enact behaviour seem sensible as ways to support patients to change their oral health behaviour, they require dedicated input from a dental professional who is motivated to help the patient. Our findings suggest that some dentists don’t see prevention as their role and may have negative feelings towards delivering prevention. Furthermore, the dentist having a negative attitude towards the patient is likely to act as a barrier to them giving oral health advice [10]. These dentists may be more likely to generalise this to patients being unwilling and unable to adopt preventive advice. Such attitudes may be affecting the provision of care at the individual and policy level to patients since in order for oral health promotion to be effective those delivering the messages need to believe in the effectiveness and efficacy of the advice that they are giving. If they don’t hold such beliefs then they are less likely to deliver effective oral health promotion. Indeed, previous research highlighted that some dental professionals felt as though their actions did little to influence patient behaviour change with regards to childhood caries, suggesting that patient motivation played an important role[35] Kay et al.,[10] conclude that oral health practitioners need a high sense of self-efficacy regarding their oral health promotion abilities. The idea of creating capability, enhancing motivation to change, and creating opportunities to enact the oral health behaviour seem like sensible strategies to support patients to change their oral health behaviour. However, future research is required to explore who is best placed to support patients in changing their oral health behaviour and in what setting this should be.

### Strengths and limitations

The discord between the patient participants’ perception of their own knowledge and value of prevention compared with theirs and the other participants’ views of patients more broadly could be explained by a number of factors. It is possible that the patient participants felt compelled to exaggerate their value on prevention since they were taking part in a focus group on this topic. Also, the patient participants may not be representative of the wider general population. Those interested in taking part in the research may be more likely to have a greater interest in oral health than the wider population. It is also possible that the policy makers, insurers and dentists may have skewed views on the publics’ attitude toward prevention as they assume patients should place the highest value on their oral health care and by not showing explicit valuing of this that they do not value prevention. However, this view fails to take into account the numerous other factors that patients have to contend with and weigh up when prioritising their oral health. The negative views of patients held by the other stakeholder groups may also be influenced by their professional role; a policy maker’s job is to aim their policies and guidance at the lowest common denominator -those most at risk - and the dentists are speaking anecdotally about patients, so it is the most extreme (and in this case the most negative) cases that stand out. The age of the data also needs mentioning; the interviews were conducted between 2016 and 2017, however, since dental contracts have changed little, it is most likely that the barriers discussed here still remain for all stakeholders involved. Indeed, it is possible that the barriers discussed here have become exacerbated in recent years with the NHS dentistry crisis [36, 37], especially when considering current issues around access to and availability of NHS dental appointments [38]. It is also important to note that our sample included only dentists and no other members of the dental team. This decision was made for several reasons. Firstly, patients are more likely to visit the dentist than any other member of the dental team. It is not yet the norm for all patients to regularly visit a hygienist or therapist in England. Secondly, prevention is a large part of a hygienist’s role and so wouldn’t have enabled the same exploration of barriers to delivering prevention as from a dentist’s perspective. We recognise that it is likely that including dental hygienists, dental therapists and dental nurses would have offered another viewpoint on the delivery of prevention. Recent research[35] does capture the viewpoint of these dental team members with regards to caries in children, but future research should aim to explore this from an adult patient perspective. Our focus on dentists is inline with the view of dentists as team leaders of the practice, as they are the individuals who run the business (which is influenced by contracts, incentives, and altruistic motivations). Although the complexity of this perspective [39] was not fully explored, given the focus of the interviews we believe that their responses do reflect some of the complexities underlying practice delivery. An additional limitation is that our sample only included patients who attended the dentist on a regular basis (at least every 2 years). It is important to note that the inverse care law is very much in operation within dental service provision [40]. The dearth of dental provision within areas of high need is apparent. The challenge of ensuring advice and preventive care reaches those with the greatest need has been highlighted and continues to be a challenge. Strategies such a proportionate universalism and the common risk factor approach are ways in which the dental team can play a role in concert with upstream approaches to improve oral health across the population [41–43]. Despite the limitations, a strength of this research is the unique perspectives and insights offered by triangulating data from patients, dentists, policy makers and insurers. The variation in perspectives between different groups of participants shows to some extent the challenges of unpicking barriers to prevention due to the many competing views and priorities.

## Conclusion

In conclusion, the findings of this research indicate that patients’ knowledge of and priority given to prevention is variable, and that targeted oral health education could be useful in improving this. Additionally, the patient-dentist relationship can influence a patient’s knowledge and receptivity to preventive messages, as well as the value they place on it. However, even with a good understanding of the importance of prevention and a positive relationship with their dentist, patients may still lack the motivation to engage in preventive behaviours. Ways to motivate the patient and the dental team to prioritise oral health prevention need further exploration to reduce barriers to the delivery of, and patient access to oral health prevention. Addressing these barriers in the future requires a multi-faceted approach to improving dental prevention in line with the COM-B model of behaviour change and should include clear communication, education, a strong patient-dentist relationship, and strategies to increase motivation.

## Electronic supplementary material

Below is the link to the electronic supplementary material.

Additional File 1: Topic guides for each stakeholder group.

Additional File 2: SRQR checklist.

## Data Availability

The datasets used and/or analysed during the current study are available from the corresponding author on reasonable request.
