# Supplementary material for: Barriers to prevention in oral health care for english NHS dental patients: a qualitative study of views from key stakeholders
Source: BMC Oral Health. 2023 May 27;23:332. doi: 10.1186/s12903-023-03030-x (PMC10225091; doi:10.1186/s12903-023-03030-x)
Supplement: Supplementary file 1 — Additional File 1: Topic guides for each stakeholder group. [file 12903_2023_3030_MOESM1_ESM.docx]

## Topic guides for each stakeholder group

| **DENTISTS** | |
| --- | --- |
| **Questions** | **Prompts or further questions** |
| I would now like you to imagine that you are seeing a regular patient who has come for a check-up. Would you provide them with any advice on how to look after their teeth and how their lifestyle could be changed to improve their oral health (work on the wording of this) |  |
| How do you quantify whether or not you have done a ‘good job’ with a patient OR how do you measure your success? |  |
| If you want to keep up to date with current guidelines or check your treatment against the best practice guidelines how do you go about doing that? | Can you suggest any ways these can be made more usable? |
| How do you feel about the current encouragement to focus on prevention measures. | What, in the current system is facilitating achieving this?  What, in the current system is acting as a barrier to achieving this? |
| What do you feel is stopping Dentists from adopting a more preventive approach to dental care? | What would aid you in overcoming this?  *Eg. Time involved, barriers getting patients to attend, extra resources/costs required*  *Eg. Support educating patients, feedback on performance, support with extra appointments,* |
| What helps you to move from a treatment to prevention focused approach? (orientating practice) (If you feel that is what you do?) |  |
| What do you think hinders you from moving patients from a treatment to prevention focused approach? (behaviour change in patients) | What do you think helps you from moving patients from a treatment to prevention focused approach? (behaviour change in patients) |
| What have patients fed back to you on their experience of a focus on prevention? | Do patients appear to value this focus or find it unnecessary and time consuming? |
| Do you think a prevention focus is attainable or sustainable given the current dental contracts and the dental structure? | If no- why? |

| **INSURERS** | |
| --- | --- |
| **Questions** | **Prompts or further questions** |
| What do you generally feel is good and bad with the current dental care system? |  |
| What would you perceive the ideal dental care system to look like/entail? | What, in the current system is facilitating or acting as a barrier to this? |
| What influences the value/focus you (as an insurance company) place on different areas of dental health? | *Eg. Cost, accessibility to services, impact on oral health* |
| What would influence why one treatment is covered by insurance over another? | *E.g why might a general check-up not be covered but a tooth extraction is?* |
| What is stopping insurance companies from supporting/emphasising the importance of preventative dental care to a greater extent? |  |
| What would you like to be in place to support prevention? |  |
| Do you feel that dentists should be incentivised to focus more on prevention? If so, how could this be achieved? |  |

| **PATIENTS** | |
| --- | --- |
| **Questions** | **Prompts** |
| Do you make visits to the dentist and what is usually the reason for you to visit the dentist? |  |
| What would motivate you to go to the dentist more often? And what are barriers? | (Does your dentist inform you that you need to come more often?) |
| What are your experiences at the dentist? What are negative aspects of a visit and what are the positive aspects? |  |
| What does ‘treatment’ mean to you? |  |
| What does ‘prevention’ mean to you? | What is your undertstanding of prevention in oral health care? |
| Do you feel that your dentist mainly focusses on treatment or prevention? What is more important to you? | (Do you feel there is a good balance between prevention and treatment) |
| What do your dentist and you usually talk about? What advice is given? |  |
| Do you think is the task/role of the dentist to give you advice about your lifestyle. About oral hygiene? About smoking? About Alcohol? | Do you appreciate such advice? |
| What would help you to keep your mouth and teeth healthy? |  |
| What do you think needs to change to stimulate the dentist to do more on prevention. | What do you think needs to be changed with the healthcare system?  Improving oral healthcare and prevention  Should discuss barriers and facilitators |

| **POLICY MAKERS** | |
| --- | --- |
| **Questions** | **Prompts or further questions** |
| How does prevention feature in your day to day role? | *(Interviewer note: Found it necessary to define prevention to be sure that both the chairside prevention and educational type of prevention were discussed)* |
| What do you think are the main barriers to improving the oral health care provided to the population? | What are the barriers? |
| How can these barriers be addressed? | How can they be overcome?  What are the challenges in overcoming these? |
| What happens which then means you begin looking at new guideline development? Can you give me some examples? |  |
| How would a change ‘somewhere’ result in guideline development and how would this then be rolled out. |  |
| How do you think patient needs are taken into account in guideline development |  |
| What do you think are the anticipated barriers to prevention for patients? |  |
| What do you think are the anticipated barriers for dentists to prevention? And you address these in your role? |  |
| From your perspective as a xxxx what would aid a transition to a preventive dental care focus? |  |
